# Identifying optimal threshold statistics for elimination of hookworm using a stochastic simulation model

**DOI:** 10.1186/s13071-017-2256-8

**Published:** 2017-06-30

**Authors:** James E. Truscott, Marleen Werkman, James E. Wright, Sam H. Farrell, Rajiv Sarkar, Kristjana Ásbjörnsdóttir, Roy M. Anderson

**Affiliations:** 1 London Centre for Neglected Tropical Disease Research (LCNTDR), Department of Infectious Disease Epidemiology, St. Mary’s Campus, Imperial College London, W2 1PG, London, UK; 2 The DeWorm3 Project, The Natural History Museum of London, London, SW7 5BD UK; 3 Division of Gastrointestinal Sciences, Christian Medical College, Vellore, 632004 India; 4 Department of Global Health, University of Washington, Seattle, USA

**Keywords:** Soil-transmitted helminths, Mass drug administration, Elimination of transmission, Cluster randomized trials, Stochastic models, Positive/Negative predictive value

## Abstract

**Background:**

There is an increased focus on whether mass drug administration (MDA) programmes alone can interrupt the transmission of soil-transmitted helminths (STH). Mathematical models can be used to model these interventions and are increasingly being implemented to inform investigators about expected trial outcome and the choice of optimum study design. One key factor is the choice of threshold for detecting elimination. However, there are currently no thresholds defined for STH regarding breaking transmission.

**Methods:**

We develop a simulation of an elimination study, based on the DeWorm3 project, using an individual-based stochastic disease transmission model in conjunction with models of MDA, sampling, diagnostics and the construction of study clusters. The simulation is then used to analyse the relationship between the study end-point elimination threshold and whether elimination is achieved in the long term within the model. We analyse the quality of a range of statistics in terms of the positive predictive values (PPV) and how they depend on a range of covariates, including threshold values, baseline prevalence, measurement time point and how clusters are constructed.

**Results:**

End-point infection prevalence performs well in discriminating between villages that achieve interruption of transmission and those that do not, although the quality of the threshold is sensitive to baseline prevalence and threshold value. Optimal post-treatment prevalence threshold value for determining elimination is in the range 2% or less when the baseline prevalence range is broad. For multiple clusters of communities, both the probability of elimination and the ability of thresholds to detect it are strongly dependent on the size of the cluster and the size distribution of the constituent communities. Number of communities in a cluster is a key indicator of probability of elimination and PPV. Extending the time, post-study endpoint, at which the threshold statistic is measured improves PPV value in discriminating between eliminating clusters and those that bounce back.

**Conclusions:**

The probability of elimination and PPV are very sensitive to baseline prevalence for individual communities. However, most studies and programmes are constructed on the basis of clusters. Since elimination occurs within smaller population sub-units, the construction of clusters introduces new sensitivities for elimination threshold values to cluster size and the underlying population structure. Study simulation offers an opportunity to investigate key sources of sensitivity for elimination studies and programme designs in advance and to tailor interventions to prevailing local or national conditions.

## Background

The soil-transmitted helminths (STH) are a group of parasites comprising whipworm (*Trichuris trichiura*), roundworm (*Ascaris lumbricoides*) and hookworm (*Ancylostoma duodenale* and *Necator americanus).* Although not generally considered fatal, chronic, high-intensity STH infections are associated with iron-deficiency anaemia, protein malnutrition, and intellectual and cognitive impairment, especially amongst children [1]. The greatest burden of STH infection falls on socio-economically disadvantaged communities in sub-Saharan Africa, China, East Asia, and the Americas. Global estimates suggest up to 1.5 billion people are infected with STH resulting in approximately 5.2 million disability-adjusted life years (DALYs), of which the majority are attributable to hookworm [2].

The current WHO approach for STH is centred on programmes of mass drug administration (MDA), using albendazole or mebendazole. The main goal is to achieve a coverage of 75% of school-aged children (SAC) within MDA programmes by 2020 [3]. Frequency of coverage is determined by disease prevalence measured among SAC, with shorter intervals between treatment rounds for higher prevalences [4]. The aim of this strategy is to eliminate STH diseases as a public health problem (defined by a threshold of 1% medium-to-heavy infection among school children). One problem with this approach is the focus on the treatment and monitoring of children, which ignores morbidity in other age groups which represent a significant proportion of the population. Additionally, the differing age profiles of infection among the different STH diseases mean that the impact of such targeted treatment will vary considerably. For example, *Ascaris* burden tends to be concentrated in SAC whereas hookworm burdens are frequently heaviest among adults. As such, treatment of SAC has a much lower impact on hookworm disease burden than on a population with a comparable *Ascaris* burden [5, 6].

A further problem with this strategy is that it does not foresee an endpoint. MDA remains necessary to control morbidity in SAC as adults, who remain untreated, continue to contribute infectious material to the environmental reservoir. Control programmes continue to treat according to the WHO guidelines, maintaining a low level of prevalence in SAC. In recent years, discussion of the control of STH has turned to the question of whether the emphasis of the WHO strategy for STH (and schistosome) infections should shift from morbidity control to the interruption of transmission [7–9]. There is a growing body of analysis that suggests that expanding MDA coverage from pre-school aged children (Pre-SAC) and SAC to the whole community can be sufficient to break transmission of STH in most settings. The impact of community-wide coverage is particularly strong for hookworm due to the relatively heavy worm burdens in adult populations [10–12].

The recently launched DeWorm3 project aims to investigate the possibility of breaking STH transmission by leveraging the work of existing lymphatic filariasis (LF) elimination programmes [13]. As LF is also treated with albendazole (in combination with ivermectin or diethylcarbamazine) using community-wide MDA, it forms the ideal platform to build on. Such programmes typically provide 4–6 yearly rounds of MDA before prevalence is reduced to a threshold level consistent with the interruption of transmission [14]. Hence there is an opportunity at the conclusion of an LF elimination programme to continue MDA, possibly in an intensified form, to break transmission of STH in the same area. The DeWorm3 studies are structured as cluster randomized controlled trials (CRT), in line with other recent studies on transmission interruption [15]. The purpose of this paper is to address some of the key design challenges arising from such studies. We have developed an individual-based stochastic model of STH transmission within an epidemiologically-independent community [16, 17]. This is taken to be equivalent to a village in a rural setting, although its interpretation is more problematic in an urban setting. From this, we construct a simulation of the prospective study; the initial LF treatment period and the STH eradication programme. The simulation allows for the variability that arises between different communities as well as that generated by the stochasticity of demographic and epidemiological processes and the uncertainties of diagnostic and sampling strategies. A key problem with detection of elimination is that it is a long term phenomenon that requires many years to pass before it can be confirmed [17]. The simulation allows individual communities to be traced forward in time to identify the long-term ‘fate’ of populations.

The simulation allows us to examine the connection between potential elimination thresholds and the elimination or bounce-back of the parasite population within a community. We examine how the probability of achieving elimination within a community depends on the baseline prevalence of infection and community size. We also test the accuracy of a range of threshold measures to predict long-term elimination and how that accuracy depends on other aspects of study design, such as time of measurement and baseline prevalence. Within the context of potential thresholds and their accuracy, clustering is likely to play an important role. Clusters are constructed from the aggregation of individual communities and hence thresholds at the cluster level will be subject to greater uncertainty due to variation among the constituent communities. Since breaking transmission occurs at a community level, the probability of achieving elimination is also likely to depend on the constitution of clusters. We examine the impact of aggregation using the study simulation, looking at the effect of cluster size and underlying community size distribution on the probability of elimination and the ability of thresholds to detect it.

## Methods

The model system used in the current paper arises from and is set in the context of modelling work performed for the Deworm3 project. The purpose of the Deworm3 project is to test the feasibility of leveraging past LF elimination effort using a cluster randomized trial [13]. The simulation follows participating communities through an initial phase of 4 years of pre-study LF treatment, followed by 3 years of twice yearly community-wide treatment at a higher coverage during the study (see Table 1). Beyond the study end-point, treatment ends and the parasite populations in communities are allowed to evolve without intervention to ascertain the long-term fate of the parasite population.

**Table 1:** Overview of main study design and demographic parameters used in simulations. Community size distributions are described in study simulation section

Default epidemiological and study parameter values	Explanation
LF MDA programme	Annual 4 years of MDA community-wide, coverage of 0.65, 0.65, 0.40 for pre-SAC, SAC and adults, respectively [25]
STH MDA programme	Bi-annual 3 years of MDA community-wide, coverage of 0.70, 0.70, 0.60 for pre-SAC, SAC and adults, respectively [3]
Baseline prevalence	Prevalence after LF MDA programme. Prevalence between 5 and 40%
Community size distribution	Vellore, Tamil Nadu: Mean = 263 and approximate range 100–800
	Indian census data, 2001: Mean = 2680 and approximate range 50–7500
Diagnostic test	McMaster, based on two samples

### Model structure

The transmission model employed focuses on hookworm as this is the most prevalent STH species in the locations chosen for the DeWorm3 project and also potentially represents one of the most difficult to eliminate through school-based deworming, having an infection age profile that typically spans both children and adults [11]. In brief, the model is a stochastic simulation of the worm burdens of individual hosts in a population. The epidemiologically independent population unit is taken to be a village or community. Births and deaths of hosts are included and are based on a typical demography of a low-income country. Mortality rates are assumed to be independent of an individual’s infection status. In simulations, initial host ages are drawn from the equilibrium age profile implied by the demography. Acquisition of worms from the infectious reservoir is mediated through an age-dependent contact rate, leading to an appropriate age profile of infection for the parasite; host contribution to infectious material in the environment has the same age dependence. The contact rate of individuals with infectious material has an underlying gamma distribution which generates the characteristic negative binomial distribution in worm burdens seen in worm expulsion epidemiological studies [18–20]. The distribution is dynamic over time given changes in a key parameter of the distribution, the mean worm burden per host.

Sexual reproduction of the parasite in the host is incorporated, which is crucial when investigating elimination processes as STH species reproduce sexually. As the number of worms per hosts decreases, the likelihood of both sexes being present in a host for the production of fertile eggs is reduced. For sufficiently low prevalences, fertile egg production becomes too low to support the parasite population in the host population, leading to the interruption of transmission. Hence there exists a critical parasite prevalence ‘breakpoint’, above which the parasite population can sustain itself and below which it collapses to the disease-free state.

The epidemiological parameters were obtained by fitting an equivalent deterministic model to individual-level intensity data from an intervention study of hookworm control in Vellore, South India [21]. Details of the model, its fitting and validation can be found elsewhere [22]. Bayesian techniques were used for fitting a likelihood function to the data, leading to a posterior distribution for the parameters. We use samples from the posterior parameter distribution to capture the underlying epidemiological variability among communities in the simulation. However, we assume that intensity of transmission, as characterised by the reproductive number R_0_, is the key source of variability in prevalence and hence vary this independently to generate a sufficiently wide range baseline prevalences for the study.

### Study simulation

Using the community-scale model described above, we construct a simulation of an elimination study following on directly from the end of a national LF programme. The simulation is constructed from four consecutive time periods: an initial 10-year equilibration period for communities to establish endemic disease transmission; a 4-year period of LF treatment; 3 years of twice-yearly intensive community-wide treatment within the study period and a final extensive period without treatment to allow communities to achieve elimination or bounce back to endemic levels. The coverage levels for the two period of treatment are given in Table 1. The coverage levels used represent approximate mean levels for the two types of treatment regime. For LF, we have extended levels ascribed to children to adults with a drop-off to reflect the added difficulty of reaching adults. Higher levels are often quoted, but it is also the case that official figures are frequently unrealistic [23]. In the case of the elimination study, levels are based on the WHO 2020 goals of 75% MDA coverage extended to adults, but allowing again for a drop-off in coverage of adults due to non-participation [3]. This is perhaps pessimistic as an elimination study would take pains to achieve the highest levels of coverage possible.

**Fig. 1:**
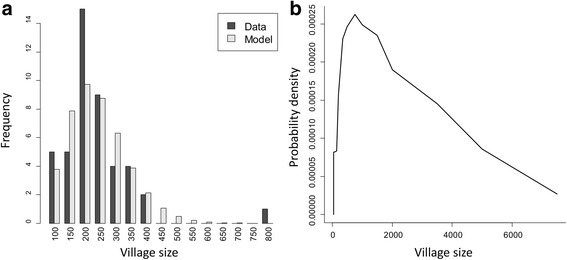
Distribution of village sizes in the Vellore study (**a**) and from the Indian census, 2001 (**b**). **a** Histogram of the Vellore data and the equivalent expectations for the fitted model (Parameters: mean = 263, aggregation parameter = 7.7. Labels give lower bounds of bins with width 50). **b** The Indian census distribution is an approximation from the number of communities in a range of size categories (mean = 2770, standard deviation = 1870)

Both the diagnostics and sampling procedures are simulated and are also stochastic processes, adding to the variance of the output. Key assumptions of the diagnostic model is that measured egg output from a host is negative binomial in distribution and that mean egg output is subject to fecundity limitation due to the number of worms present in a host [24]. Hookworm only release eggs when fertilized, so egg output requires both male and female worms present [10]. In this study, we assume that McMaster is the diagnostic method based on two independent stool samples, in agreement with the study to which the diagnostic model was fitted [21, 22]. For the population sampling, we randomly select 200 people from the entire population of a study demographic unit. The diagnostic technique and sampling method applied in this study will increase the variance in the prevalence measured and influence the distribution of the threshold statistic and its critical threshold value. The output of the simulation is used to construct the appropriate demographic study unit, if necessary, and generate a sample at a given time point and perform the diagnostic test on it. This process is repeated many times to generate a probability distribution for the sampled state of the demographic unit and the associated final state (parasites eliminated or bounced-back). In this study, we look at two types of demographic study units: single communities and clusters of communities of a given size. For single communities, we examine the sensitivity of elimination and threshold statistics to size. For clusters, we construct groups of communities of various total population sizes from underlying distributions of community sizes. We have used two sources of data that inform the community size distribution. The Vellore study, against which the model was calibrated, was conducted across 45 communities whose demography was recorded [21]. Figure 1a shows a histogram of this data along with the expectations from a negative binomial distribution with the maximum likelihood. However, communities within this study are significantly smaller than average community sizes in India (mean size 263, range 100–800). For a more representative distribution, we use data from the Indian census of 2001 to construct an approximate probability distribution, shown in Fig. 1b [25]. This distribution is characterised by a mean an order of magnitude higher, at 2680, and ranges from 50 to 7500. For each of the village size distributions, clusters were constructed by randomly accumulating communities so that their sizes fell into predetermined ‘bins’ and the statistics of each bin were analysed to generate the dependence on size. The mean sizes of bins were in intervals of 500 from 500 up to 10,000 with boundaries at the mid-points. Sample sizes from the clusters were 200 individuals and elimination was declared if all constituent communities in a cluster achieved parasite elimination.

In the following section, we first look at the overall response of prevalence in communities as they progress through LF treatment, the study itself and then on to parasite bounce back or elimination. We compare several different candidate threshold measures for their ability to differentiate at the end of the study between eliminating and recovering parasite populations. We examine the dependence of the probability of achieving elimination on community size and R_0_. The R_0_ range present in our sets of parameter values is chosen to approximately match the baseline prevalences measured in the Vellore study (5–45%). As such, it is hard to say how much clusters constructed from this population will be generalizable. For this reason we also filter results by measured baseline prevalence ranges. This helps to minimize the dependence of the results on the background distribution of R_0_ and also represents a more intuitive measure of transmission intensity.

As the prevalence is reduced substantially after such intense MDA, a threshold is needed to differentiate at the end of the study between simulations achieving interruption of transmission and simulations recovering to the endemic state (bounce-back). The quality of threshold measures in distinguishing between interruption of transmission and bounce back is reflected by the positive and negative predictive values (PPV and NPV, respectively) [26]. In the current context, the positive predictive value is defined as the proportion of eliminations detected by the threshold statistic that result in long-term eliminations. Correspondingly, the negative predictive value is the proportion of bounce-backs detected by the statistic that result in recovery to endemic infection states. The predictive value measures are attractive in this context as they factor in the prevalence of communities that eliminate. As such, they estimate the probability of true elimination based on based on information available from the threshold test alone.

In the context of an elimination study, it can be argued that a high PPV is most important. A key requirement of an elimination programme is that it results in some degree of certainty as to whether the goal has been achieved. A low PPV value indicates that communities tested as eliminations are likely to bounce back eventually, leading to treatment programmes being terminated early before transmission has been broken. In contrast, low values of NPV encourage programme managers to assume elimination has not been achieved at the end point, when it has. This may incur an economic penalty from continued treatment, but does not affect the epidemiological effectiveness of the programme or study.

## Results

**Fig. 2:**
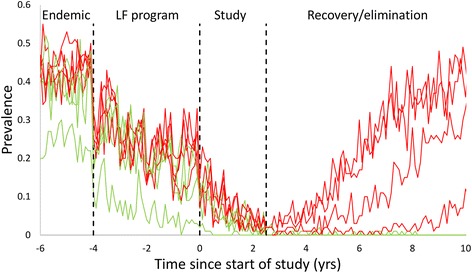
Time series of measured prevalence in a selection of individual communities within the stochastic simulation. Vertical lines indicate the four distinct regions of the simulation; endemic behaviour, LF treatment period, the duration of the study and the post-study period without MDA treatment. *Red* and *green* lines indicate communities that ultimately bounce back or eliminate, respectively

Figure 2 shows the measured prevalence of a selection of communities over time, with the long-term fate of each indicated by line colour. The combination of the LF programme and subsequent treatment within the study brings all communities to a low prevalence state. At the end of the study, individual communities resolve into either an elimination or recovery trajectory. Eliminating communities remain at low prevalences but parasites may persist in the population for another 5 or more years. Bounce-back communities display considerably more variability. Prevalences vary between individual communities due to differences in epidemiological parameters and within a community over time due to the variation in which individuals are sampled and variability in the egg output from individuals as well as the diagnostic test performance. Note that the individual rounds of treatment are very hard to identify within the variability between individual measurements.

**Fig. 3:**
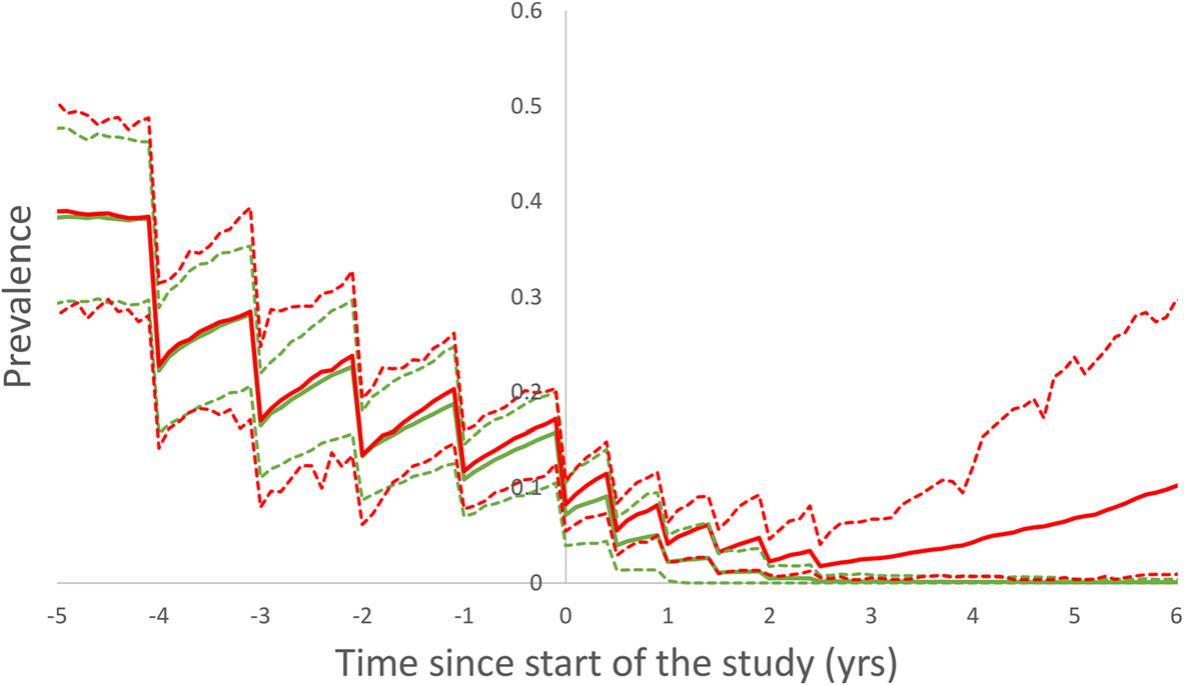
Summary statistics for measured prevalences across communities with baseline prevalence in the range 10–20% going to elimination (*green*) and bouncing back (*red*). Solid lines represent mean values and broken lines the 95% prediction interval

Figure 3 shows the mean measured prevalence and 95% prediction intervals for prevalence, averaged across communities that eliminate or bounce back, respectively. The impact of differing transmission intensities among communities has been controlled for by including only those with a baseline prevalence between 10 and 20%. The two groups are indistinguishable during the LF programme and only start to differ during the elimination study. Variability across eliminating communities is low at the study end point and continues to drop with time. Among bounce-back communities, variance is initially larger and increases in the years directly following the end of the study. The ‘entanglement’ of the measured prevalences of the two classes of communities indicates that it may be difficult to identify a good threshold to distinguish them.

### Village level results

**Fig. 4:**
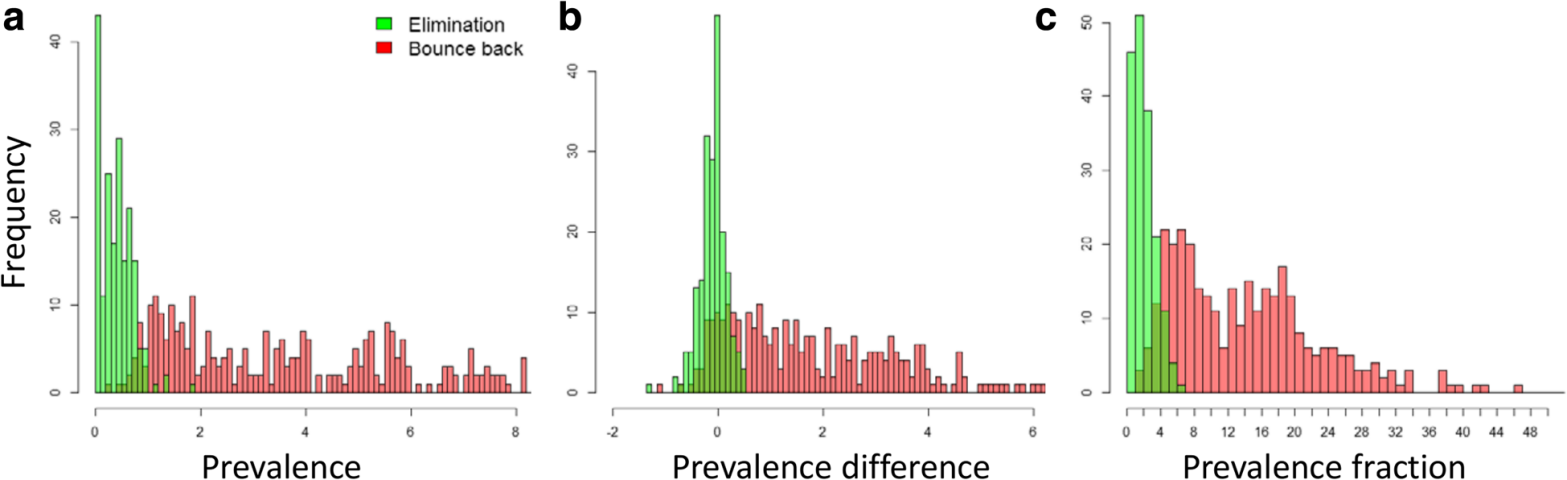
Histograms for three possible post-study threshold statistics: **a** measured prevalence at 1 year post-study; **b** prevalence difference between 1 year and 3 months post-study; and **c** the ratio of prevalence at 1 year post-study to baseline prevalence. Values from eliminating and rebounding communities are *green* and *red*, respectively. Results represent 1000 model iterations

In this section, we consider the quality of several possible threshold statistics for elimination and their sensitivity to aspects of study design and epidemiology. For a threshold statistic to be effective, it must be possible to choose a critical value that can discriminate between the two outcomes of interest. This can to some extent be determined by eye from distribution of the statistic across multiple measurements. Figure 4 shows the distributions of three potential end-point statistics, as applied to individual communities, and shaded according to whether each village went on to achieve elimination of infection (green) or bounce back to endemic levels (red) in the long term after treatment was ended.

For both prevalence measured one year after study end-point and, to a lesser extent, end-point prevalence as a fraction of baseline, the distribution for eliminating and re-establishing communities are partially distinguishable. For prevalence, the threshold value lies somewhere close to 1%, while for the baseline fraction, the threshold is close to 5% of baseline value. Prevalence difference between 3 months after and 1 year after study end is clearly less differentiated, with the range of prevalence differences from eliminating village being shared by a reasonable proportion of re-establishing communities (Fig. 4c). This is a consequence of the ‘noisiness’ of prevalence values at study end in comparison to the absolute prevalence. Comparison of two prevalence serves to double the variance, obscuring any trend on the underlying mean. This is on top of the extra cost and logistical effort of measuring the prevalence twice.

Both the overall probability of elimination and the ability of a threshold to detect elimination at the end point are strongly dependent on the baseline prevalence. Baseline prevalence serves as a proxy for transmission intensity, so the range of R_0_ values associated with each baseline prevalence range is also shown. A problem with looking at the statistics of communities selected from a population with a wide range of transmission intensities is that the probability of elimination and threshold quality will depend on the background (prior) distribution of R_0_. However, the R_0_ distribution associated with the parameterizing dataset may not be generalizable to other populations. By choosing from a narrow range, we minimize the impact of this variability. The association between community size and elimination is well established for micro-parasitic diseases in the concept of critical community size (first identified by Bartlett [27]). In these cases, small communities generate low numbers of infectious individuals which are prone to stochastic fade-out, even when R_0_ is greater than 1. In the current model, this effect is complicated by the existence of a deterministic breakpoint separating the endemic and disease-free states, as described above.

**Fig. 5:**
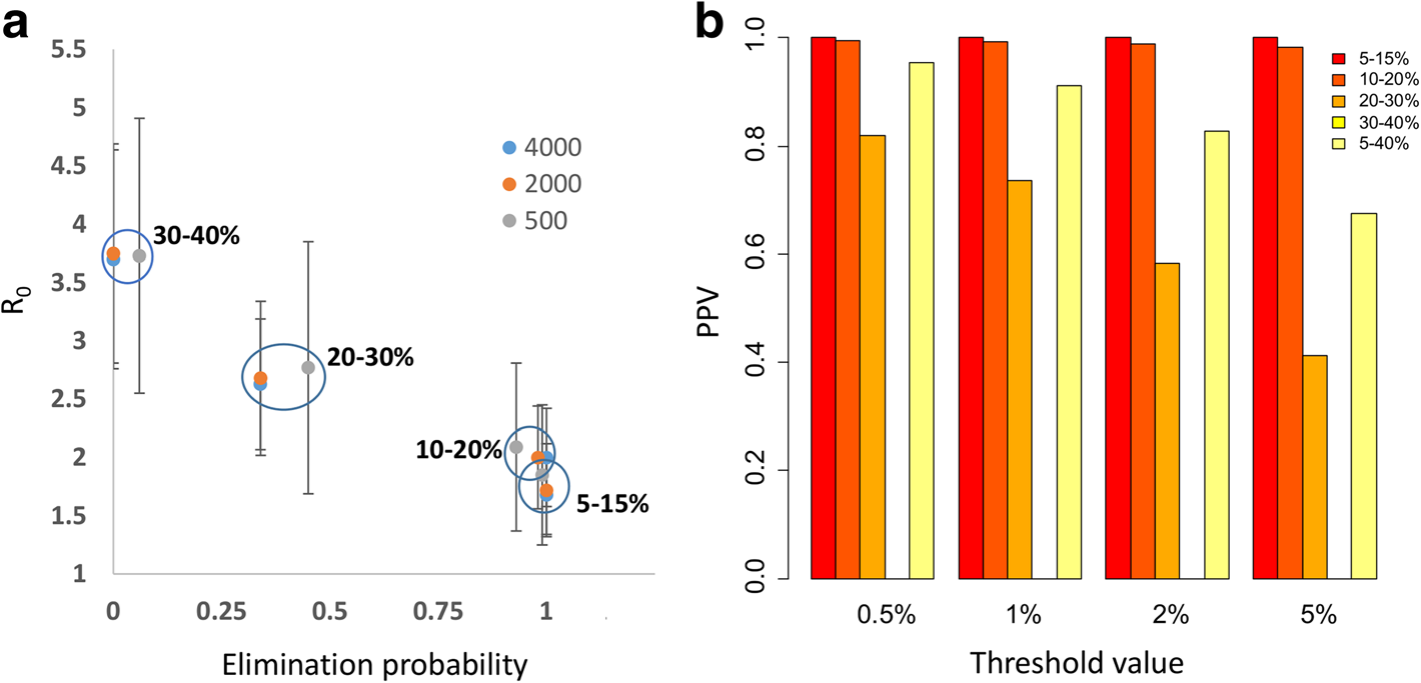
**a** Probability of elimination for communities with different baseline prevalence ranges and across a range of village population sizes. Error bars show 2 standard deviations for the R_0_ ranges of different baseline prevalence limits (indicated by circles). **b** PPV values for a range of elimination thresholds and baseline prevalences

Figure 5a shows the relationship between probability of elimination and baseline prevalence, community size and R_0_. There is a clear association between baseline prevalence and probability of elimination. Prevalences below 20% are almost certain to achieve elimination while above 30% baseline, elimination is not possible with the prior treatment and study design used. Probability of elimination is only weakly correlated with community size. Results from communities of size 2000 and 4000 are indistinguishable in terms of elimination. Smaller communities of size 500 are 5–10% more likely to eliminate for a given baseline prevalence. This indicates that, at least for communities of 500 individuals or more, the elimination process is dominated by the deterministic breakpoint rather than size-dependent effects.

Figure 5b illustrates the effectiveness of a range of prevalence threshold values, taken 1 year post study, to detect elimination. In this case, we have a sample size of 200, which is just large enough to measure a 0.5% prevalence threshold. As the absolute probability of elimination decreases with increasing baseline prevalence (and R_0_), the positive predictive value of all thresholds also decreases. For baseline prevalences less than 20%, PPV remains well above 95% for all thresholds examined. However, under these conditions, the probability of elimination is very likely. For baselines of 20–30%, with a probability of elimination around 40%, a threshold of 2% or less is required to achieve a PPV greater than 60%. PPV values for baseline range 30–40% are not available since no communities from this range achieve elimination. More representative of a population of communities, if communities are drawn from a broad range of baseline prevalences (5–40%), a threshold of 2% is capable of distinguishing elimination with a PPV of above 80%.

### Cluster level results

**Fig. 6:**
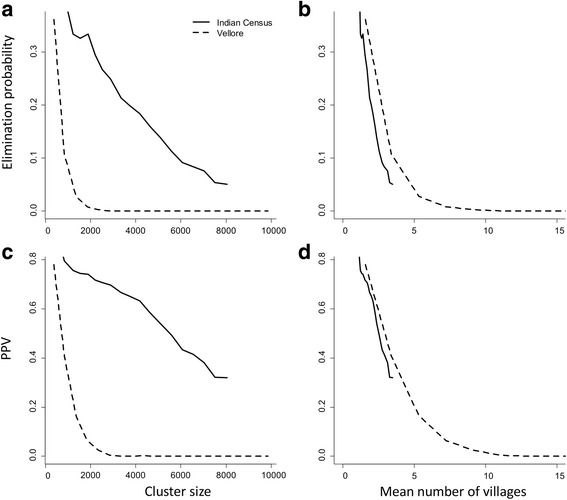
Impact of cluster size and composition on probability of elimination (**a** and **b**) and threshold PPV (**c** and **d**). Probability of elimination and PPV are plotted against cluster size (**a** and **c**) and mean number of communities (**b** and **d**), respectively. Prevalence threshold is set at 2%, one year post study, with baseline prevalence range of 5–40% and sample size of 200 individuals

Figure 6a shows statistics for clusters of various sizes, constructed from communities taken from the two distributions described. Clusters are constructed from communities with a range of baseline prevalences from 5 to 40%. Probability of elimination is strongly dependent on cluster size and the underlying distribution of village sizes. When constructed from the smaller communities in the Vellore distribution, the probability of elimination drops rapidly to zero by about a cluster size of 2000 individuals. In the case of the Indian Census communities, probability of cluster elimination also decreases with size, but more slowly. Figure 6b indicates that major determinant of this behaviour is the number of communities in a cluster. Since elimination within a cluster requires elimination within all constituent communities, the probability of elimination in a cluster might be expected to have an approximately exponential dependence on the number of communities if the probability of elimination were the same across communities. Some of the remaining discrepancy between clusters from the two village size distributions can be accounted for by the differences in their ranges. As shown in Fig. 5a, small communities are more likely to achieve elimination than large ones, due to the increased importance of stochastic variability. Clusters constructed from the Indian census data are less likely to contain small communities and will on average be less likely to be driven to elimination by treatment, as seen in Fig. 6b. Very similar effects are at play in the dependence of PPV on cluster size (Fig. 6c, d). PPV was calculated for a threshold of 2% prevalence at one year post-study. A sample of individuals taken from a cluster effectively samples from all the constituent communities and the measured prevalence is a weighted mean of the individual village prevalences. As such, the sampled prevalence can be below the threshold while individual communities may be above it, increasing the likelihood of a failure to eliminate and reducing the PPV. The greater the number of constituent communities, the more likely that one or more communities will fail to eliminate, leading to a drop in PPV with cluster size and number of communities. The cluster size effect for NPV is the opposite. Any collection of communities within a cluster that tests negatively against the elimination threshold will contain communities with prevalences above the cluster mean and hence more likely to bounce back to endemicity. As a result, the whole cluster will fail to eliminate as predicted. NPV values across all cluster sizes tend to be very close to 1.

**Fig. 7:**
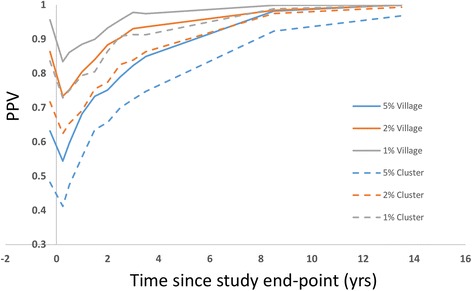
Sensitivity of PPV to time since end of study for communities (mean = 2770) and clusters of size (3–5000) individuals. Sample size is 200 individuals and the overall probability of elimination is approximately 27%

The quality of the threshold statistic is also sensitive to the time at which it is recorded. Figure 7 shows the dependence of PPV on time since study end. The time dependence in PPV closely reflects that of the prevalence mean and prediction interval shown in Fig. 3. As the parasite populations recover in the bounce-back communities, the two groups become more easily distinguished and the PPV improves. The difference between village and cluster level results is not large in this case as the mean size of the two groupings is relatively close. The bounce back rate for PPV is approximately exponential with a half-life of approximately 3 years. Hence threshold quality improves markedly within 1–2 years of the end of the study.

### Sensitivity of diagnostic testing

The number of samples taken from individuals within a sample will vary according to local practice or available resources. WHO protocols are not specific, but standard practice is 2 samples [28]. Throughout the paper we have assumed two samples, but here we investigate the sensitivity to number of samples taken. Increasing sampling will naturally lead to greater diagnostic sensitivity to prevalence and an increase in PPV and NPV scores. Our analysis shows that using one sample performs substantially poorer than using two or more samples, however there is no benefit in using more than two samples (Table 2).

**Table 2:** Impact of sensitivity of diagnostics on PPV and NPV values. Rows represent different numbers of independent McMaster test done on each individual in the sample. Baseline prevalence range 5–40%

Number of samples	Threshold quality (PPV / NPV)			
	0.5%	1%	2%	5%
1	0.97/0.96	0.91/0.99	0.81/1	0.65/1
2	0.99/0.91	0.97/0.98	0.88/1	0.74/1
3	0.99/0.87	0.97/0.95	0.87/1	0.72/1
4	1/0.87	0.98/0.95	0.88/1	0.73/1

## Discussion

The precise assessment of the infectious state of a population is complicated by the many sources of variation and uncertainty. The underlying stochasticity of infection and demographic processes is compounded by the process of constructing a sample and the sensitivity of the diagnostic tools. For the large populations involved in CRTs and MDA programmes, there is the additional factor of the variability within the population; in this case, the variation in the demographic structures and epidemiological rates across different communities. The methodological approach adopted in this paper has endeavoured to include these sources of uncertainty, their interactions with each other and their magnitude, as captured from data collected from a large STH control study [21]. The power of this simulation model is that it provides the opportunity to examine a large range of covariates related to the design of studies and programmes and link them to long term end states such as elimination or recovery. To study these phenomena directly in the field would require decades and throw up a number of obvious ethical issues. For example, programme managers are obliged to treat participants who are identified as infected. Treating participants in the control group could possibly dilute the differences between two arms and is therefore undesirable.

Within the current paper, we have addressed the issue of the quality of potential threshold statistics and their sensitivity to design features such as the construction of the study demographic units and the time of measurement and epidemiological aspects like baseline prevalence ranges. The time series shown in Figs. 2 and 3 illustrate the key features of the variability in measurements. They show that variability around the mean prevalence after the end point of the study is relatively small for communities that reach elimination. In contrast, for communities that bounce back, variability in prevalence post study grows rapidly and takes at least ten years to recovery to endemic levels. Figure 2 shows that communities that are bouncing back may spend a number of years at very low prevalence levels. The overlap of these two distributions is clear in the histograms of the three possible statistics, categorised by final state, shown in Fig. 4. Both prevalence difference and prevalence ratio have more overlap, and hence less resolution, than a single measure of prevalence; the prevalence difference particularly so. This is partly explained by the fact these two statistics are constructed from two separate prevalences and hence the combined variance is the sum of the variance at each time-point. The difference in prevalence at two time points is commonly used to quantify the effects of a programme, but is predicated on the assumption that correlations between successive measurements can be used to ‘neutralise’ the variance between individuals. However, close to elimination, variance to mean ratios are particularly large, swamping any benefit achieved.

The idea of identifying a valid threshold for elimination is reinforced by the fact that sexual reproduction of the worms within a host leads to a breakpoint prevalence threshold below which there are insufficient fertilised females to maintain the transmission cycle. For micro-parasitic diseases, there is generally no equivalent threshold. This difference is reflected in the impact of community size on elimination. For communities larger than 500 individuals, the probability of elimination is largely independent of population size. This contrasts with the micro-parasitic diseases, where critical community size is a key determinant of the persistence of infection in a population [27]. Independence from community size is also a consequence of the transmission model used, in which transmission intensity does not scale with community size.

The ability to bring about elimination in a community is clearly dependent on its baseline prevalence, which in turn is a function of transmission intensity (R_0_) and past LF treatment [29]. For baseline prevalences above 30%, elimination in the current treatment context is not possible with the coverages assumed in this study. For prevalences where elimination is possible, thresholds of 2% or less are required to achieve PPV scores of greater than 50%. Results shown in Fig. 5 are based on sample sizes of 200. PPV values can be marginally improved with larger samples.

Cluster-level results differ markedly from those for individual communities. Due to economies of scale and logistic considerations, studies and monitoring and evaluation for programmes are usually based on clusters or regions composed of a number of communities. As shown in Fig. 6, probability of elimination within a cluster and the PPV of thresholds drops of rapidly with increasing number of constituent communities. This reflects the fact that, at least within the current model framework, elimination is a property of individual communities rather than whole regions. By viewing elimination at the level of cluster or region, some detail is inevitably lost. In scenarios in which all communities are have transmission intensities low enough for the study or programme to achieve elimination with certainty, the size and constitution of clusters will not be important. However, in scenarios in which elimination is not certain across all communities, the quality of thresholds can be very sensitive to the size of clusters and the distribution of community sizes.

Our analysis indicates that there are no significant benefits to applying more than two samples per individual when determining infection prevalence. However, it is important to note that the parameters used in the diagnostic model were estimated from an extremely conscientious testing procedure within a trial context [21]. It is likely that when diagnostics tests are performed in the context of national programmes, the diagnostic process will be of a lower quality due to the large volume of samples, financial restrictions, administrative challenges and inadequate training of personnel. It will depend on health economic considerations to ensure a cost-effective approach. New diagnostic technologies such as qPCR may improve the accuracy as a measure of the presence of infection in less controlled settings [30, 31].

A number of issues remain to be explored. The choice of hookworm with its broadly flat age-intensity profile, along with community-wide MDA and sampling, minimizes the impact of age structure. Both *Ascaris* and *Trichuris* tend to have infection much more concentrated in school-aged children and this will have a big impact on where elimination thresholds will lie, particularly as SAC are the usual focus of STH monitoring. In such a scenario, threshold prevalences sampled from SAC are likely to be considerably higher. Given a study design with SAC-focused MDA, as recommended by WHO, a further complication would arise from the age profile of host contributions to the reservoir. If the majority of infectious material is contributed by hosts outside the targeted group, the impact of treatment will be greatly reduced. Unfortunately, very little is known about this aspect of the transmission cycle.

Along with the size and constitution of clusters, the distribution of transmission intensities among constituent communities has a strong impact on the probability of elimination and its detection, as shown. The distribution in this case is characteristic of the dataset used for the parameterization of the model and can’t be generalised to other scenarios. The range of transmission intensities should be chosen to match the baseline prevalences of a particular study and prior LF treatment programme for the threshold and PPV values to be appropriate. Indeed, a clear use of a study simulator is to determine an appropriate distribution of transmission intensities matching baseline prevalences in the light of known prior LF coverages. This is in addition to a knowledge of the size distribution of the communities. A related question is what corresponds to a community as represented in our model. In a rural setting, this refers to a village, but it is less clear in an urban setting. It is also unclear to what extent neighbouring communities are epidemiologically independent. People within one village may have a lot of contact with another village and perhaps within a different cluster. For STH, this may be important given that individuals can deposit transmissions stages via defaecation on travels between communities. However, it should be noted that the range of spatial correlation for hookworm has been observed to be less than 100 m [32, 33]. These additional correlating processes will presumably reduce the variance of measurements within clusters. Measures of migration and movement are necessary to resolve these questions and it is hoped that the DeWorm3 project will contribute to a better understanding of these effects.

A further potential issue is the structure of the transmission model itself. Models of STH transmission generally employ a single environmental reservoir of infectious material. While these models generally perform well on validation, they have not been tested rigorously at low prevalence yet [22]. There is evidence of heterogeneity at the household level for STH and this could lead to different dynamics at low prevalences [33]. Again, it is hoped that the detailed monitoring within the DeWorm3 project, among other ongoing studies, will inform modelling in this prevalence regime.

As illustrated by the analyses reported in this paper, setting a threshold prevalence for elimination needs careful consideration. For a given PPV, baseline prevalence, prior treatment, cluster design and community size distribution all play a part. The design and implementation of such studies and the elimination programmes that arise from them are time-consuming and costly processes. Simulations such as the one presented in this paper offer a guide to this process and illustrate the key types of data necessary.

## Conclusions

Breaking transmission is increasingly a goal for NTDs. In practice, efforts to break transmission and to confirm the resulting state of elimination require huge resources and take place over an extended time scale. Model simulations offer a chance to investigate and gain insight into the elimination process ‘in silico’, informing the process of program design. The results from the current modelling show that prevalence thresholds have the potential to determine whether elimination is successfully reached. The ability of biannual MDA to achieve elimination and the quality of thresholds to detect it (as measured by PPV) is highly sensitive to baseline prevalence, with thresholds of 2% or less required for PPV value greater than 50%. Baseline prevalence is sensitive both to the intrinsic transmission intensity in a population and also to its prior history of treatment, so information about patterns of past treatment are essential.

The breaking of transmission is a phenomenon with a geographical scale determined by the nature of epidemiological mixing in the population. We have identified this scale as the village or community, but this may vary with the social structure of a population. Elimination programs are concerned with breaking transmission in populations comprising many of these basic units. Our results show that breaking transmission and detecting it in large populations is sensitive to the both the size and demographic constitution of these populations. Much of the sensitivity is accounted for by the total number villages within a population, with larger numbers leading to lower probabilities of elimination and lower PPV values for the thresholds to detect it. Our work indicates that baseline prevalence, past treatment history and the social structure of a population are key indicators of the success of an elimination program and should be the focus of data collection.

## Abbreviations

LF: Lymphatic filariasis
MDA: Mass drug administration
NTD: Neglected tropical diseases
Pre-SAC: Pre-school aged children
SAC: School-aged children
STH: Soil-transmitted helminths
WHO: World Health Organization
